# Diet quality is associated with obesity and hypertension in Australian adults: a cross sectional study

**DOI:** 10.1186/s12889-016-3714-5

**Published:** 2016-10-01

**Authors:** Katherine M. Livingstone, Sarah A. McNaughton

**Affiliations:** Deakin University, Institute for Physical Activity and Nutrition, School of Exercise and Nutrition Sciences, Melbourne Burwood Campus, 221 Burwood Highway, Geelong, VIC 3125 Australia

**Keywords:** Diet quality, Obesity, Hypertension, Obesity-related hypertension, Adults

## Abstract

**Background:**

Poor diet, characterized by a low diet quality score, has been associated with greater prevelence of obesity and hypertension. However, the evidence is inconsistent across diet quality scores and by sex. The aim was to investigate the relationship between diet quality and obesity and hypertension.

**Methods:**

Adults (*n* = 4908; age 45.2 ± 0.24 years) were included from the cross-sectional Australian Health Survey 2011–2013. Two 24-h dietary recalls were used to derive the dietary guideline index (DGI) and recommended food score (RFS). Logistic regression investigated relationships between diet quality score and odds ratio of obesity, hypertension and obesity-associated hypertension.

**Results:**

In the highest tertile of DGI, but not RFS, individuals were less likely to be obese (men: OR 0.64, CI: 0.45, 0.92, *P*-trend = 0.014; women: 0.68, 0.48, 0.96, *P*-trend = 0.025) and to have central adiposity (men: 0.68, 0.48, 0.97, *P*-trend = 0.030; women: 0.53, 0.37, 0.77, *P*-trend = 0.001) compared with the lowest tertile.

Men, but not women, in the highest tertile of DGI and RFS were less likely to be hypertensive (DGI: 0.56, 0.37, 0.85, *P*-trend = 0.006; RFS: 0.62, 0.41, 0.94, *P*-trend = 0.021) compared with the lowest tertile. In men with obesity, but not normal weight men or women, those in the highest tertile of DGI were less likely to be hypertensive (0.53, 0.36, 0.78, *P*-trend = 0.001) compared with the highest tertile.

**Conclusions:**

Higher diet quality, as estimated using DGI, was associated with lower odds ratio of obesity in men and women. Odds ratio of hypertension was lower in men, but not women, with a high diet quality score compared with a low score, while obesity-associated hypertension was only associated with diet quality score in men with obesity. Longitudinal studies are needed to evaluate whether diet quality predicts risk of obesity and hypertension.

**Electronic supplementary material:**

The online version of this article (doi:10.1186/s12889-016-3714-5) contains supplementary material, which is available to authorized users.

## Background

Non-communicable diseases, such as hypertension, and obesity are among the leading causes of premature death [1]. An unhealthy diet is a major modifiable behavioral risk factor in the development of obesity and hypertension [1]. Although a variety of dietary recommendations for the management and prevention of obesity and hypertension have been proposed [2], evidence is inconsistent and varies between measures of dietary intake used. Furthermore, with 65 to 75 % of the incidence of hypertension directly related to obesity [3], the role of diet in obesity-associated hypertension is an important consideration for the development of dietary guidelines.

Studies to date have focused primarily on the relationship between single nutrients and foods and obesity and hypertension [4, 5]. However, given foods are not consumed in isolation, an increasing body of research is investigating total diet [6], or diet quality, and its impact on disease [7]. Diet quality scores have most widely been assessed in relation to cardiovascular disease and cancer [8].

Although there have been studies on obesity and hypertension [7, 9, 10], methodologies for deriving diet quality scores vary [11] and findings differ by sex and score used [10]. In a cross-sectional study, the Dietary Guideline Index (DGI) was inversely associated with central adiposity in men [7], while in a prospective study, the Framingham nutritional risk score was inversely associated with obesity in women [12]. Similarly, the Healthy Diet Indicator was inversely associated with hypertension in men [10], while the Recommended Food Score (RFS) was inversely associated with blood pressure in both men and women.

As emphasized by the Dietary Patterns Methods Project [13], comparison between diet quality methodologies across different health outcomes is needed to strengthen the evidence base for future policy development. Diet quality scores based on adherence to the Australian Dietary Guidelines (ADG;﻿ e.g. DGI) measure overall diet, whereas those based on adherence to recommended food intakes (e.g. RFS) reflect healthy food intakes only. Given that previous research has shown that RFS predicts mortality, whereas non-RFS (based on “unhealthy” foods) does not [14], an evaluation of the effectiveness of an overall diet score and a healthy-component focused score is warranted to identify which diet quality methodology is associated with odds ratio of obesity and hypertension. Furthermore, to date, no study has evaluated both DGI and RFS in relation to obesity-related hypertension.

The present study adapted the DGI and RFS for use in the Australian Health Survey (AHS), a nationally-representative cross-sectional study of Australian households [15]. The aims of this analysis were to i) investigate the relationship between two diet quality scores and obesity-related outcomes and hypertension and i) identify whether two diet quality scores were associated with obesity-related hypertension in Australian adults.

## Methods

### Subjects and study design

The present analyses were based on adults (19–85 y) from a subset of the latest (2011/13) AHS [15]: the Australian National Nutrition and Physical Activity Survey (NNPAS; *n* = 4908). As described elsewhere [15], the AHS is a population-based survey that sampled households in urban and rural areas across all states and territories in Australia. Dietary intakes and habits were estimated in the NNPAS using two 24-h dietary recalls and a food habits and attitudes questionnaire. Anthropometric and blood pressure measures were collected by trained interviewers at selected clinics or home visits.

### Study measures

#### Obesity-related outcomes

Body weight (BW; k﻿g), height (cm) and waist circumference (WC; cm) measurements were measured on a voluntary basis by trained interviewers using digital scales, a stadiometer and a metal tape respectively. Pregnant women were not measured. Subjects were encouraged to remove their shoes and any heavy clothing prior to having measurements taken. Body mass index (BMI) was derived using Quetelet’s metric (kg/m^2^). Standard cut offs for BMI and WC were applied: underweight/normal weight: BMI < 25 kg/m^2^; overweight: BMI ≥ 25 kg/m^2^ and <30 kg/m^2^; obese: BMI ≥ 30 kg/m^2^; and central adiposity: WC > 102 cm (men) and >88 cm (women) [16].

#### Blood pressure and hypertension

Blood pressure measurements were voluntary. Systolic (SBP) and diastolic blood pressure (DBP) measurements were taken on the left arm, unless there was a prohibitive reason such as an injury. Interviewers undertook two blood pressure readings using an automated blood pressure monitor in which systolic and diastolic pressures were displayed. Individuals were categorized as non-hypertensive (blood pressure <140/90 mmHg) and hypertensive (≥140/90 mmHg). Data on hypertensive medication were not recorded [15].

#### Socio-demographic characteristics

Socio-demographic characteristics were collected in the NNPAS via interviewer-administered questionnaires. Smoking was defined as ex﻿-smoker, cu﻿rrent smoker and never smoked. Education status was operationalized as low (some high-school or less), medium (high-school or some high-school and/or certificate/diploma) and high (tertiary qualification) [15]. Urban or rural location was defined as major city, inner rural or other [15]. Physical activity (PA) was assessed according to i) meeting PA guidelines (150 min of PA per week and 150 min of PA over 5 or more sessions per week) and ii) time spent sedentary (minutes per day). Female life stage was operationalized as: never having menstruated, experiencing menopause or post-menopause. Further details are provided elsewhere [15].

#### Dietary intake

An automated, multiple-pass, 24-h dietary recall was used to provide quantitative information on foods and beverages consumed on the day prior to interview based on the Agricultural Research Service of the United States Department of Agriculture Automated Multiple-Pass Method [17]. The interview was divided into five phases: quick list description of food and beverages consumed the previous day, prompt to remember any omitted foods, information on time and eating occasion, further details and a final probe to recall any omitted foods of beverages [15]. A second 24-h recall, via telephone interview, was collected at least 8 days after the first interview. Nutrient intakes were derived from the 24-h recalls using the Australian Supplement and Nutrient Database 2011–13, developed by Food Standards Australia New Zealand [18]. Information on type of milk consumed, usual daily intake of fruit and vegetables and use of salt were collected in the NNPAS survey [15]. Energy misreporting was calculated as the ratio of energy intake to predicted total energy expenditure (using sex and age-specific equations for a range of weight status, assuming a PA level of "low active" PA level ≥ 1.4 and PA level < 1.6) [19].

#### Dietary guideline index

The DGI is a food-based score designed to reflect the diet quality of subjects according to compliance with the 2013 ADG for Australian adults [20]. The DGI used in the present study was based the DGI-2013 [21], and thus comprised a total of 23 items (see Additional file 1: Table S1), but was adapted from use in food frequency questionnaires (FFQ) to use in the present 24-h recalls. DGI scores ranged between 0 and 130, with a higher score indicating better diet quality. Each item was scored out of ten (zero indicating the guideline was not met). Items with two sub-components were scored out of five. Cut-offs used to obtain the maximum score for each component were tailored to age and sex-specific food-based recommendations outlined in the ADG [22]. Proportionate scores were derived where intakes fell between the maximum and minimum soring criteria for all items except discretionary foods, saturated and unsaturated fat, salt, sugar and alcohol [23, 24].

A food variety score was estimated based on the variety of foods consumed within the five core food groups: fruit, vegetables, meat or meat alternatives, dairy and cereals [25]. Foods scored 1 point if consumed above a cut off (>15 g/d for beverages and >20 g/d for foods), analogous to the RFS [26], and variety was estimated within each core food group by summing scores for each food group and dividing by the total number of foods within each core food group. Scores were summed across the five core food groups and multiplied by two to create a score out of 10 [27]. Salt intake was assessed using two questions from the food habits and attitudes questionnaire: frequency of adding salt during cooking and during a meal and saturated fat intake was scored against the ratio of trimmed meat to total meat intake and low fat milk to total milk intake. Sub-components of cereal, meat and alternatives and fluid intake captured information on the ratio of wholegrain bread to total bread, lean meat to total meat and water to total fluid intake, respectively. Total beverage intake included milk and soy beverages, smoothies, fruit and vegetable juices, low calorie cordials, low calorie soft drinks, water, tea and coffee. Flavored milk drinks, fruit drinks, high-sugar soft drinks, cordials and alcohol were not included in total beverage intake due to the associations with dental caries, weight gain and diabetes [28], but were included as “Discretionary foods”. “Discretionary foods”, defined as energy-dense foods and drinks that are not essential to nutrition, included sugar-sweetened beverages, sweet and salty snacks and confectionary, cakes and pastries, high-fat processed meats and dishes, pies, fried foods, ice cream and other dairy desserts, cream, butter and spreads and alcoholic beverages [29]. Cut-offs for discretionary food intakes were sourced from the ADG companion resource for educators [22]. Unsaturated fats included intakes of nuts, seeds and margarine, while added sugar included confectionary, jam, marmalade, honey, syrup and sugar-sweetened beverages.

#### Recommended food score

The RFS is a food-based diet variety score calculated based on the frequency of consumption of foods from five core food groups: fruits (6 items), vegetables (9 items), wholegrains (4 items), lean meats and alternatives (2 items) and low-fat dairy (2 items). Scores ranged between 0 and 23, with a higher score indicating a better diet quality. Scoring was based on a method by Kant and Graubaud [30] for use with 24-h recall data, where foods were assigned a score of 1 if they were consumed above the minimum amount threshold: 15 g/d for non-beverages and 30 g/d for beverages. Two additional methods for calculated RFS were tested. The first of these methods assigned a score of 1 for each recommended food if consumption was ≥0.5 servings over the 2 day recalls [27]. The second additional method was based on sex-specific median cut offs for consumers, where a score of 1 was allocated if consumption was above the median cut off [31].

#### Statistical analyses

Participants were excluded from the present analyses if they i) were pregnant and/or breastfeeding ii) had missing data for outcomes and covariates ii) only 1 day of dietary recall. All analyses were conducted for men and women separately. Variables were tested for skewness and kurtosis and were log transformed if not normally distributed. BW, BMI, and WC were log transformed prior to analysis. Tertiles of diet quality score were selected as the optimum methodology for evaluating variations in diet quality score based on maximizing power from the sample size and previous literature [32]. Linear regression and chi squared tests were used to test for significant differences in participant characteristics across tertile of diet quality score and in diet quality score between sexes for categorical and continuous variables respectively. To answer our primary research question (Does the odds ratio of obesity, central adiposity and hypertension vary by tertile of diet quality score?), multi-variable-adjusted logistic regression analyses were performed. To assess these relationships in continuous outcomes multiple linear regression analyses were performed. Analyses were adjusted for age (continuous), smoking (categorical), physical activity﻿﻿ (﻿whether met PA recommendations; binary), education (categorical), urban or rural location ﻿(categorical), energy misreporting﻿ (ratio of energy intake to predicted total energy expenditure; ﻿continuous), dieting or atypical dietary intake on day of reporting (categorical) and female life stage (categorical; women only). Hypertension-related outcomes were further adjusted for BMI (continuous). To answer our secondary research question (Does the odds ratio of hypertension by tertile of diet quality score vary according to obesity status of the population?), multi-variable-adjusted logistic regression analyses stratified by BMI status (normal weight vs. overweight or obese) and by central adiposity (no central adiposity vs. central adiposity) were performed. Analyses were adjusted for the same covariates mentioned above with the exception of BMI.

Data were analyzed using Stata (version 14; StataCorp., College Station, TX, USA) using survey weightings for analyzing complex survey data to account for the survey design. These weightings were specifically designed to account for bias associated with those who volunteered to complete the second day of dietary recalls (64 %; *n* = 7735). Weighting was calibrated to align with independent benchmarks in designated categories of sex by age and area of usual residence [15]. *P* < 0.05 was considered statistically significant.

## Results

**Fig. 1:**
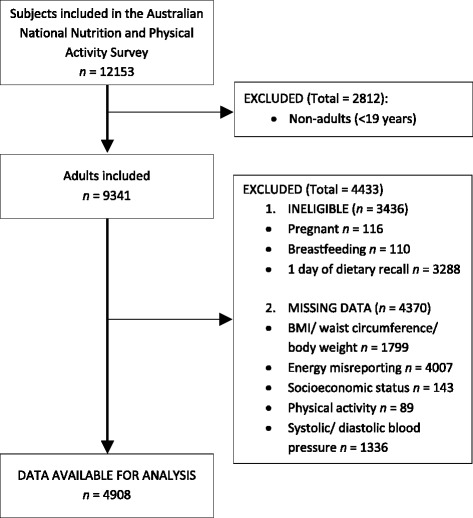
Flow diagram of subjects included in the cross-sectional analysis of the Australian National Nutrition and Physical Activity Survey

As summarized in Fig. 1, a total of 4908 individuals (age 45.2 ± 0.24 years) were included in the present analyses (men: *n* = 2346; women: *n* = 2562). Mean DGI and RFS were higher in women compared with men (*P* < 0.001; Additional file 1: Table S1 and Table S2 respectively). For DGI components and sub-components, women scored better than men on 12 out of 23 items (Additional file 1: Table S1), while for RFS, women scored better on five out of 21 components (Additional file 1: Table S2).

Compared with those in the lowest tertile, men and women in the highest tertile of DGI were older and smoked less (*P* < 0.001). Among men, those in the highest tertile of DGI, had higher levels of education and more individuals met PA recommendations (*P* < 0.001). Among women, PA (*P* = 0.004) and SBP (*P* = 0.007) were higher in the highest tertile of DGI, while time spent sedentary was lower (*P* = 0.007; Table 1). Results were similar for RFS (Additional file 1: Table S3).

**Table 1:** Participant characteristics according to tertile (T) of Dietary Guideline Index (DGI) (*n* = 4908)^a^

	Overall	Dietary Guideline Index			*P*-trend^b^
		T1	T2	T3	
Men					
*n*	2346	865	755	726	-
DGI score	79.2 ± 0.47	64.1 ± 0.38	80.5 ± 0.21	95.7 ± 0.40	<0.001
Age, y	45.2 ± 0.24	43.2 ± 0.70	46.6 ± 0.81	46.3 ± 0.87	0.018
Education, %					
Low	19.9	24.6	18.4	15.8	<0.001
Medium	54.1	56.2	56.2	49.4	
High	26.0	19.2	25.4	34.9	
Smoking, %					
Current smoker	18.8	28.6	14.9	11.2	<0.001
Ex-smoker	34.9	32.4	39.0	33.6	
Never smoked	46.3	39.0	46.2	55.2	
Physical activity					
Sedentary behavior, min/d	363 ± 6.3	375 ± 8.5	343 ± 9.7	370 ± 10.9	0.60
Meet recommendations, %	47.2	40.4	47.5	55.0	<0.001
BMI, kg/m^2 c^	27.7 ± 0.15	27.9 ± 0.30	27.4 ± 0.26	27.6 ± 0.27	0.53
BMI category, %					
Underweight/normal weight	30.8	30.2	31.2	31.1	0.25
Overweight	42.9	39.5	44.8	44.9	
Obese	26.4	30.3	24.1	24.0	
Waist circumference, cm^c^	97.7 ± 0.38	98.6 ± 0.73	97.1 ± 0.64	97.1 ± 0.66	0.22
Systolic blood pressure, mmHg	125.5 ± 0.59	126.5 ± 1.16	125.6 ± 0.73	124.4 ± 0.78	0.14
Diastolic blood pressure, mmHg	77.1 ± 0.31	77.6 ± 0.59	77.0 ± 0.55	76.6 ± 0.48	0.15
Women					
*n*	2562	757	885	920	-
DGI score	80.9 ± 0.48	64.7 ± 0.48	80.4 ± 0.17	94.6 ± 0.29	<0.001
Age, y	46.9 ± 0.30	42.2 ± 0.79	47.0 ± 0.63	50.5 ± 0.73	<0.001
Education, %					
Low	27.2	27.1	25.6	28.8	0.038
Medium	43.0	47.4	45.4	37.1	
High	29.8	25.5	29.1	34.0	
Smoking, %					
Current smoker	14.4	21.9	13.5	9.1	<0.001
Ex-smoker	28.0	27.7	27.9	28.2	
Never smoked	57.7	50.4	58.6	62.7	
Physical activity					
Sedentary behavior, min/d	313 ± 4.0	336 ± 10.4	312 ± 8.3	296 ± 6.7	0.007
Meet recommendations, %	43.9	36.8	44.2	49.3	0.004
BMI, kg/m^2 c^	27.0 ± 0.20	27.2 ± 0.40	26.7 ± 0.27	27.1 ± 0.33	0.86
BMI category, %					
Underweight/normal weight	44.8	43.3	44.8	46.0	0.84
Overweight	29.6	31.2	30.1	27.7	
Obese	25.7	25.6	25.1	26.4	
Waist circumference, cm^c^	87.3 ± 0.47	87.6 ± 0.94	86.9 ± 0.72	87.4 ± 0.72	0.96
Systolic blood pressure, mmHg	119.5 ± 0.46	116.9 ± 0.99	120.1 ± 0.85	121.1 ± 0.95	0.007
Diastolic blood pressure, mmHg	76.0 ± 0.32	75.7 ± 0.68	76.0 ± 0.54	76.2 ± 0.54	0.58

^a^ Values represent means ± SD or percentages

^b^ Linear regression and chi squared tests were used to test for significant differences between groups in continuous and categorical variables, respectively

^c^ Data were log-transformed prior to analyses; values represent the geometric mean ± SD

### Obesity

As summarized in Tables 2 and 3, in the highest tertile of DGI, compared with the lowest, men and women were less likely to be overweight or obese (*P*-trend = 0.014 and *P*-trend = 0.025 respectively) and have central adiposity (*P*-trend = 0.030 and *P*-trend = 0.001 respectively). Although the direction of the relationship was the same for RFS, no significant relationships between RFS and odds ratio of overweight or obesity, or central adiposity were observed. Results were comparable when estimated according to the two alternative RFS (data not shown).

**Table 2:** Multi-variable-adjusted odds ratio and 95 % CI of obesity and hypertension per tertile (T) of Dietary Guideline Index (DGI) in a nationally representative sample of Australian men (*n* = 2346) and women (*n* = 2562)

	Dietary guideline index			*P*-trend^a^
	T1	T2	T3	
Overweight and obese				
Men^b^				
Cases, *n*	587	547	501	-
Proportion, %	71.3	70.0	69.8	-
Crude	1.0	0.94 (0.68, 1.31)	0.92 (0.65, 1.32)	0.68
Model 1	1.0	0.85 (0.60, 1.19)	0.84 (0.59, 1.20)	0.32
Model 2	1.0	0.71 (0.49, 1.02)	0.64 (0.45, 0.92)	0.014
Women				
Cases, *n*	404	505	551	-
Proportion, %	58.1	56.9	55.1	-
Crude	1.0	0.95 (0.71, 1.28)	0.89 (0.65, 1.21)	0.43
Model 1	1.0	0.88 (0.65, 1.20)	0.75 (0.55, 1.02)	0.06
Model 2	1.0	0.83 (0.58, 1.19)	0.68 (0.48, 0.96)	0.025
Central adiposity				
Men				
Cases, *n*	508	461	430	-
Proportion, %	61.0	58.3	59.4	-
Crude	1.0	0.89 (0.65, 1.22)	0.94 (0.68, 1.30)	0.66
Model 1	1.0	0.77 (0.56, 1.05)	0.83 (0.59, 1.19)	0.29
Model 2	1.0	0.66 (0.48, 0.93)	0.68 (0.48, 0.97)	0.030
Women				
Cases, *n*	494	597	606	-
Proportion, %	69.5	66.4	63.7	-
Crude	1.0	0.87 (0.61, 1.23)	0.77 (0.55, 1.07)	0.12
Model 1	1.0	0.71 (0.50, 1.02)	0.56 (0.41, 0.79)	0.001
Model 2	1.0	0.70 (0.47, 1.06)	0.53 (0.37, 0.77)	0.001
Hypertension^c^				
Men				
Cases, *n*	225	187	139	-
Proportion, %	27.0	23.6	19.1	-
Crude	1.0	0.84 (0.64, 1.10)	0.64 (0.45, 0.91)	0.011
Model 1	1.0	0.68 (0.51, 0.92)	0.53 (0.37, 0.76)	0.001
Model 2	1.0	0.67 (0.49, 0.93)	0.52 (0.35, 0.78)	0.001
Model 3	1.0	0.71 (0.52, 0.99)	0.56 (0.37, 0.85)	0.006
Women				
Cases, *n*	125	190	192	-
Proportion, %	17.6	21.1	20.2	-
Crude	1.0	1.26 (0.86, 1.85)	1.19 (0.75, 1.88)	0.48
Model 1	1.0	1.03 (0.67, 1.59)	0.81 (0.49, 1.34)	0.36
Model 2	1.0	1.08 (0.69, 1.69)	0.86 (0.50, 1.49)	0.54
Model 3	1.0	1.13 (0.73, 1.75)	0.90 (0.52, 1.53)	0.62

^a^ Data were analyzed using multi-variable-adjusted logistic regression. Model 1: adjusted for age and education; Model 2: additionally adjusted for smoking, physical activity, urban or rural location, energy intake misreporting and information on whether a participant was on a diet and whether their dietary recall was typical of their habitual consumption; Model 3: additionally adjusted for body mass index

^b^ Analyses were based on *n* = 2324 due to exclusion of underweight men

^c^ Hypertension was defined as blood pressure ≥140/90 mmHg

**Table 3:** Multi-variable-adjusted odds ratio and 95 % CI of obesity and hypertension per tertile (T) of Recommended Food Score (RFS) in a nationally representative sample of Australian men (*n* = 2346) and women (*n* = 2562)

	Recommended food score			*P*-trend^a^
	T1	T2	T3	
Overweight and obese				
Men^b^				
Cases, *n*	631	566	439	-
Proportion, %	70.0	70.1	71.4	-
Crude	1.0	1.01 (0.77, 1.32)	1.07 (0.77, 1.49)	0.70
Model 1	1.0	0.87 (0.66, 1.15)	0.79 (0.54, 1.15)	0.19
Model 2	1.0	0.87 (0.65, 1.19)	0.86 (0.56, 1.32)	0.43
Women				
Cases, *n*	454	540	426	-
Proportion, %	56.0	59.9	52.8	-
Crude	1.0	1.17 (0.93, 1.49)	0.88 (0.66, 1.18)	0.41
Model 1	1.0	1.06 (0.86, 1.42)	0.72 (0.53, 0.97)	0.037
Model 2	1.0	1.24 (0.93, 1.66)	0.92 (0.65, 1.32)	0.70
Central adiposity				
Men				
Cases, *n*	542	470	389	-
Proportion, %	59.2	57.9	62.8	-
Crude	1.0	0.95 (0.71, 1.26)	1.16 (0.90, 1.51)	0.33
Model 1	1.0	0.77 (0.58, 1.03)	0.78 (0.58, 1.06)	0.07
Model 2	1.0	0.80 (0.58, 1.10)	0.89 (0.62, 1.28)	0.41
Women				
Cases, *n*	542	622	533	-
Proportion, %	65.4	68.1	65.1	-
Crude	1.0	1.13 (0.83, 1.54)	0.99 (0.70, 1.40)	0.96
Model 1	1.0	0.99 (0.70, 1.41)	0.75 (0.52, 1.08)	0.14
Model 2	1.0	1.10 (0.74, 1.64)	0.95 (0.62, 1.44)	0.82
Hypertension^c^				
Men				
Cases, *n*	226	181	143	-
Proportion, %	24.7	22.3	23.2	-
Crude	1.0	0.88 (0.64, 1.19)	0.92 (0.67, 1.26)	0.53
Model 1	1.0	0.67 (0.48, 0.93)	0.55 (0.38, 0.79)	0.001
Model 2	1.0	0.73 (0.51, 1.04)	0.62 (0.41, 0.92)	0.017
Model 3	1.0	0.73 (0.51, 1.06)	0.62 (0.41, 0.94)	0.021
Women				
Cases, *n*	162	172	173	-
Proportion, %	19.5	18.8	21.1	-
Crude	1.0	0.96 (0.63, 1.46)	1.10 (0.80, 1.53)	0.56
Model 1	1.0	0.74 (0.47, 1.18)	0.71 (0.49, 1.04)	0.08
Model 2	1.0	0.76 (0.46, 1.24)	0.77 (0.50, 1.17)	0.22
Model 3	1.0	0.73 (0.45, 1.19)	0.75 (0.49, 1.14)	0.18

^a^ Data were analyzed using multi-variable-adjusted logistic regression. Model 1: adjusted for age and education; Model 2: additionally adjusted for smoking, physical activity, urban or rural location, energy intake misreporting and information on whether a participant was on a diet and whether their dietary recall was typical of their habitual consumption; Model 3: additionally adjusted for body mass index

^b^ Analyses were based on *n* = 2324 due to exclusion of underweight men

^c^ Hypertension was defined as blood pressure ≥140/90 mmHg

Linear regression analyses identified that among men, DGI was inversely associated with BMI (*P* = 0.004), BW (*P* = 0.040) and WC (*P* < 0.001), whereas no significant relationships were identified in women (Additional file 1: Table S4). RFS was not significantly associated with any obesity outcomes.

### Hypertension

Men in the highest tertile of DGI and RFS, were less likely to be hypertensive (*P*-trend = 0.006 and *P*-trend = 0.021 respectively) compared with the lowest tertile. No relationships between diet quality and hypertension were identified in women. The pattern of results were comparable when estimated according to alternative RFS (data not shown).

Linear regression analyses confirmed that DGI and RFS were significantly inversely associated with SBP (*P* = 0.006 and *P* = 0.009 respectively) and RFS was significantly inversely associated with DBP (*P* = 0.005) in men but not women (Additional file 1: Table S4).

### Obesity-related hypertension

**Fig. 2:**
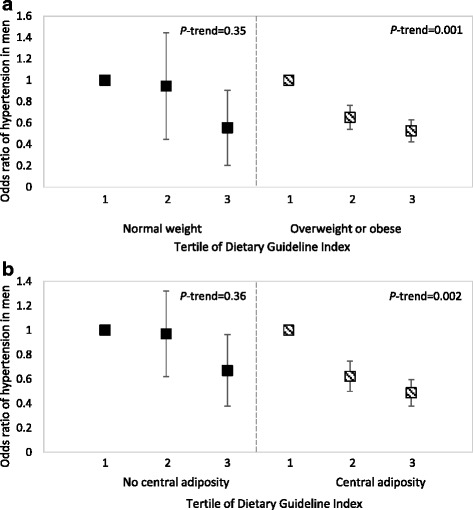
Plot of odds ratio (OR) of hypertension by tertile of Dietary Guideline Index (DGI) in men included in the Australian Health Survey stratified by **a** BMI status and **b** central adiposity, determined by multivariable logistic regression, with 95 % confidence intervals. Analyses were adjusted for age, education level, smoking, physical activity, urban or rural location, energy intake misreporting, information on whether a participant was on a diet and whether their dietary recall was typical of their habitual consumption. Tertile 1 of the DGI represented the lowest (unhealthiest) score and was used as the reference (OR = 1). Underweight men (*n* = 22) were excluding from the BMI stratification analysis. Normal weight (*n* = 630) was defined as BMI ≥ 18.5 and <25 kg/m^2^; Overweight or obese (*n* = 1694) was defined as BMI ≥ 25 kg/m^2^. Central adiposity (*n* = 1480) was defined as waist circumference >102 cm and no central adiposity (*n* = 866) as waist circumference ≤102 cm

When stratified by overweight and obesity, in the highest tertile of DGI, the odds ratio of hypertension was lower in men who were overweight and obese (OR 0.53, CI: 0.35, 0.78; *P*-trend = 0.001) and men with central adiposity (OR 0.49, CI: 0.31, 0.76; *P*-trend = 0.002) compared with the lowest tertile of DGI (Fig. 2). No significant differences were observed for normal weight men and men with no central adiposity. Furthermore, no findings were significant for RFS in men and for any associations in women.

## Discussion

The aims of this study were to investigate the relationship between two measures of diet quality and obesity and hypertension in a nationally-representative sample of Australian adults. The main findings are that a higher diet quality score, as estimated using DGI, was associated with lower odds ratio of being overweight or obese in men and women. We also identified that a higher diet quality score, using both DGI and RFS, was strongly inversely associated with hypertension, but in men only. Moreover, we observed that DGI was associated with lower odds ratio of hypertension in men who were overweight and obese only. These findings highlight the differing potential of these two diet quality scores to estimate relationships with disease, how their applicability may vary between men and women and how diet quality may play a role in obesity-associated hypertension.

Although the evidence in relation to diet quality score and obesity is inconsistent [33], our findings are comparable with previous studies, where relationships have been identified in either men or women only [7, 12, 34] or both [9]. After a 16-year follow up, the Framingham Nutrition Study identified that women with lower diet quality were more likely to become overweight or obese compared with those with higher diet quality [12]. A further study identified that after a 13-year follow up, men with a higher diet quality score were less likely to be obese, but that results were weaker or non-significant in women [34]. In a previous cross-sectional, nationally representative study in Australian adults the DGI was inversely associated with central adiposity in men and no association with obesity was observed in women [7]. To date, our study is the second to evaluate the relationship between DGI and obesity in the Australian population and is the first to compare two diet quality scores in this context [7]. Although both the current study and that of McNaughton et al. estimated diet quality using the DGI adapted to the Australian population, differing methodologies (FFQ vs 24-h recall) may account for the sex discrepancies.

Our findings in relation to diet quality and hypertension are confirmed by previous studies [7, 10, 30, 35]. The most widely cited of these studies is the Dietary Approaches to Stop Hypertension (DASH) study, in which a diet high in fruit and vegetables and low-fat dairy products and low in processed meat, reduced SBP and DBP by 5.5 and 3.0 mmHg more, respectively, than the control diet in men and women [35]. More recently, in two studies in men only, lower diet quality score has been associated with greater odds ratio of hypertension compared with a higher score [7, 10]. The reason for a lack of association in women in the present study may be due the higher diet quality scores observed in women, which suggests that women were more health-motivated [36] and thus may have changed their diet to reduce their risk of hypertension.

Our findings demonstrate that diet quality may play an important role in lowering the odds ratio of hypertension in obese individuals. Interestingly, given the difference in ORs between tertiles of DGI, our data suggest that even a moderately higher diet quality score (from T1 to T2) is associated with a markedly lower odds ratio of hypertension. With the management of diet and lifestyle identified by the *Obesity Society* and the *American Society of Hypertension* as an important focus for the treatment of obesity-related hypertension [37], our findings suggest that improving diet quality could be a valuable strategy. Nonetheless, further research into the mechanism of obesity-associated hypertension are needed to better understand the role that diet quality may have.

The present study compared odds ratios of obesity and hypertension using two diet quality scores: DGI, a 23-item component score which considers the total diet and includes both “healthy” (13 DGI foods/beverages that’s consumption is recommended) and “unhealthy” (10 DGI foods/beverages that’s consumption should be limited) components (DGI) and the RFS, which is based on 5 recommended food groups only. Both scores provided a valuable insight into how poorly Australians are adhering to the ADG. As observed previously [38], no associations between RFS and obesity-related outcomes were observed. These findings may be due to the use of 24 h recalls, which may not adequately capture the dietary exposures used in the RFS. Nevertheless, a recent systematic review and meta-analysis concluded that diet diversity scores, which share similarities with the RFS, were not associated with obesity in cross-sectional studies [39]. This suggests that the disparity in inclusion of “unhealthy” food groups between scores may be the reason for a lack of significant findings. Importantly, associations between obesity and “unhealthy” foods included in the DGI, such as salt [40], are well established and thus the lack of inclusion of these food groups in the RFS may be critical for its ability to accurately estimate relationships with obesity. Further studies are warranted to better understand the applicability of different diet quality scores to different health outcomes.

### Strengths and limitations

The present study has a number of strengths. Given this study was conducted in a large, nationally representative sample of Australian adults, our results are generalizable to the wider Australian population. Importantly, we derived two independent diet quality scores, which facilitated a comparison between an overall DGI and a RFS. The strengths of using these dietary assessment tools is that that they capture intakes of food groups consistent with Australian policy on dietary recommendations, thereby providing substantiating evidence for their population benefits. Diet quality scores used in the present study were the first scores to be adapted to a 24-h recall using age and sex-specific cut-offs, thus providing a resource for future applications of diet quality scores to 24-h recalls.

A limitation of this study was that due to its cross-sectional design we were unable to infer any causal relationships between diet quality score and obesity and hypertension. In addition, while our analyses were adjusted for multiple confounders, including energy misreporting, we cannot discount the possibility of residual confounding. Prospective studies are warranted to determine whether higher diet quality scores will predict lower odds ratio of obesity and hypertension in the future. Although diet quality scores have many practical advantages, they focus on selected food groups and therefore do not account for the overall correlated structure of dietary patterns. Furthermore, adaption of the DGI-2013 for use in the present study may have introduced differences compared with previous studies. However, these are likely to be minimal, given that the consistency of food groupings, criteria and approach to scoring of the DGI used in the present study were consistent with the original methodology and the ADG.

### Implications of findings

Our findings have two major implications for the development of future dietary guidelines. Firstly, we demonstrated the importance of inclusion of “unhealthy” components in dietary guidelines for effectively capturing odds ratio of obesity and hypertension. Secondly, we highlighted the potential for diet quality in the management of obesity-related hypertension. The present study thus provides substantiation for the ADG and incentive for their future implementation and translation.

## Conclusions

Higher diet quality, as estimated using DGI, was associated with lower odds ratio of obesity in both men and women. Among men only, diet quality was associated with lower odds ratio of hypertension and obesity-related hypertension. Longitudinal studies are warranted to evaluate whether diet quality predicts risk of obesity and hypertension in men and women.

## Additional file

Additional file 1: Diet quality score component scores, participant charactertistics and continuous outcomes. (DOCX 34 kb)

## Abbreviations

ADG: Australian dietary guidelines
AHS: Australian health survey
BW: Body weight
BMI: Body mass index
DBP: Diastolic blood pressure
DGI: Dietary guideline index
FFQ: Food frequency questionnaire
NNPAS: Australian national nutrition and physical activity survey
PA: Physical activity
RFS: Recommended food score
SBP: Systolic blood pressure
