# Supplementary material for: Diet quality is associated with obesity and hypertension in Australian adults: a cross sectional study
Source: BMC Public Health. 2016 Oct 1;16:1037. doi: 10.1186/s12889-016-3714-5 (PMC5045600; doi:10.1186/s12889-016-3714-5)
Supplement: Additional file 1: — Diet quality score component scores, participant charactertistics and continuous outcomes. (DOCX 34 kb) [file 12889_2016_3714_MOESM1_ESM.docx]

**Table S1** Dietary Guideline Index (DGI) component scores and percentage of Australian men (n=2346) and women (n=2562) meeting dietary guidelines

| DGI components^1^ | DGI component score^2^ | | % meeting guideline^3^ | |
| --- | --- | --- | --- | --- |
|  | Men | Women | Men | Women |
| 1. Food variety | 3.3 ± 0.05 | 3.6 ± 0.05*** | 0.0 | 0.0 |
| 2. Vegetables | 4.6 ± 0.09 | 5.0 ± 0.10** | 10.3 | 10.5 |
| 3. Fruit | 5.5 ± 0.11 | 5.7 ± 0.10 | 31.2 | 26.1** |
| 4. Cereal (total) | 5.4 ± 0.08 | 5.2 ± 0.08 | 0.5 | 0.6 |
| 4a. Serves per day | 3.2 ± 0.04 | 2.7 ± 0.04*** | 15.6 | 12.6 |
| 4b. Mostly wholegrain | 2.1 ± 0.07 | 2.3 ± 0.07* | 35.9 | 43.6*** |
| 5. Meat and alternatives (total) | 7.9 ± 0.07 | 7.9 ± 0.07 | 27.5 | 25.9 |
| 5a. Serves per day^3^ | 3.3 ± 0.05 | 3.2 ± 0.05 | 28.0 | 26.1 |
| 5b. Mostly lean^3^ | 4.6 ± 0.03 | 4.7 ± 0.03 | 80.9 | 85.5** |
| 6. Total dairy and alternatives | 5.4 ± 0.11 | 4.4 ± 0.09*** | 18.8 | 8.2*** |
| 7. Fluid intake | 8.2 ± 0.07 | 8.8 ± 0.05*** | 36.0 | 38.2 |
| 7a. Serves per day | 3.7 ± 0.05 | 4.2 ± 0.03*** | 43.4 | 44.7 |
| 7b. Mostly water | 4.5 ± 0.04 | 4.6 ± 0.03** | 73.6 | 77.0* |
| 8. Limit discretionary foods | 2.3 ± 0.13 | 3.0 ± 0.12** | 23.4 | 30.0** |
| 9. Limit saturated fat | 8.6 ± 0.07 | 8.8 ± 0.07 | 51.5 | 56.0* |
| 9a. Mostly trimmed meat | 4.0 ± 0.05 | 4.2 ± 0.04* | 61.4 | 66.6* |
| 9b. Mostly low-fat milk | 4.6 ± 0.04 | 4.6 ± 0.04 | 84.5 | 86.2 |
| 10. Moderate unsaturated-fat | 8.9 ± 0.08 | 7.6 ± 0.13*** | 89.2 | 76.2*** |
| 11. Limit added salt | 5.8 ± 0.10 | 6.1 ± 0.10* | 20.5 | 21.0 |
| 11a. During cooking | 2.6 ± 0.07 | 2.6 ± 0.07 | 38.3 | 40.8 |
| 11b. Added at the table | 3.2 ± 0.06 | 3.4 ± 0.06** | 49.4 | 53.5 |
| 12. Limit extra sugar | 6.1 ± 0.13 | 6.8 ± 0.14** | 61.1 | 67.6** |
| 13. Limit alcohol | 8.1 ± 0.11 | 9.1 ± 0.10*** | 81.4 | 91.1*** |
| Total DGI | 79.2 ± 0.47 | 81.0 ± 0.44** | - | - |

^1^ DGI sub-components ranged from 0-5, while the remaining components were 0-10 and the total DGI-2013 scores range was 0-130

^2^ Values represent the survey weight adjusted mean ± SE; Data were analyzed using linear regression, where significantly different from men is denoted by * <0.05, ** P<0.01, *** P<0.001.

^3^ Those with a maximum DGI component score were considered meeting the guideline. Data were analyzed using linear regression, where significantly different from men is denoted by * <0.05, ** P<0.01, *** P<0.001.

| RFS components^1^ | RFS component score^2^ | |
| --- | --- | --- |
|  | Men | Women |
| 1. Fruit |  |  |
| 1a. Pomme | 0.37 ± 0.01 | 0.41 ± 0.01 |
| 1b. Berry | 0.10 ± 0.01 | 0.17 ± 0.01** |
| 1c. Citrus | 0.25 ± 0.02 | 0.27 ± 0.01 |
| 1d. Stone | 0.13 ± 0.01 | 0.18 ± 0.01** |
| 1e. Tropical and subtropical | 0.36 ± 0.02 | 0.39 ± 0.01 |
| 1f. Other | 0.28 ± 0.02 | 0.33 ± 0.01** |
| 1g. Juice | 0.23 ± 0.01 | 0.19 ± 0.01* |
| 2. Vegetables |  |  |
| 2a. Green and brassica | 0.47 ± 0.02 | 0.48 ± 0.02 |
| 2b. Legumes | 0.03 ± 0.01 | 0.04 ± 0.01 |
| 2c. Carrot and root | 0.31 ± 0.02 | 0.33 ± 0.01 |
| 2d. Starchy | 0.40 ± 0.02 | 0.43 ± 0.02 |
| 2e. Tomato and tomato products | 0.36 ± 0.02 | 0.37 ± 0.01 |
| 2f. Peas and beans | 0.21 ± 0.01 | 0.22 ± 0.01 |
| 2g. Other | 0.46 ± 0.01 | 0.48 ± 0.02 |
| 3. Whole grains |  |  |
| 3a. Bread and bread rolls | 0.45 ± 0.02 | 0.46 ± 0.02 |
| 3b. High fiber cereals, pasta and brown rice | 0.50 ± 0.02 | 0.49 ± 0.01 |
| 4. Lean meats and alternatives |  |  |
| 4a. Chicken and turkey | 0.28 ± 0.01 | 0.27 ± 0.02 |
| 4b. Fish | 0.24 ± 0.02 | 0.27 ± 0.01 |
| 4c. Eggs, nuts, seeds, tofu and beans | 0.46 ± 0.02 | 0.44 ± 0.02 |
| 5. Low-fat dairy |  |  |
| 5a. Milk | 0.44 ± 0.01 | 0.54 ± 0.02*** |
| 5b. Yoghurt and cheese | 0.13 ± 0.01 | 0.22 ± 0.02*** |
| Total RFS | 6.4 ± 0.10 | 7.0 ± 0.10*** |

**Table S2** Recommended Food Score (RFS) component scores and percentage of Australian men (n=2346) and women (n=2562) meeting dietary recommendation

^1^ RFS components ranged from 0-1 and the total RFS scores ranged was 0-21

^2^ Values represent the survey weight adjusted mean ± SE; Data were analyzed using linear regression, where significantly different from men is denoted by * <0.05, ** P<0.01, *** P<0.001.

**Table S3** Participant characteristics according to tertile (T) of Recommended Food Score (RFS; n=4908)

|  | Overall | Recommended Food Score | | | P-trend^1^ |
| --- | --- | --- | --- | --- | --- |
|  |  | T1 | T2 | T3 |  |
| Men |  |  |  |  |  |
| *n* | 2346 | 940 | 816 | 590 |  |
| RFS | 6.4 ± 0.10 | 3.4 ± 0.07 | 7.0 ± 0.05 | 10.6 ± 0.09 | **<0.001** |
| Age, y | 45.2 ± 0.43 | 41.3 ± 0.56 | 45.8 ± 0.78 | 50.7 ± 0.83 | **<0.001** |
| Education |  |  |  |  |  |
| Low | 19.9 | 21.3 | 17.9 | 20.4 | 0.07 |
| Medium | 54.1 | 56.2 | 56.1 | 47.9 |  |
| High | 26.0 | 22.6 | 25.9 | 31.7 |  |
| Smoking, % |  |  |  |  |  |
| Current smoker | 18.8 | 28.2 | 13.5 | 11.1 | **<0.001** |
| Ex-smoker | 34.9 | 29.5 | 39.2 | 37.6 |  |
| Never smoked | 46.3 | 42.3 | 47.3 | 51.3 |  |
| Physical activity |  |  |  |  |  |
| Sedentary behavior, min/d | 363 ± 6.3 | 371 ± 9.3 | 362 ± 9.5 | 355 ± 14.5 | 0.34 |
| Meet recommendations, % | 47.2 | 40.3 | 50.8 | 53.1 | **0.002** |
| BMI, kg/m^2^ | 27.7 ± 0.15 | 27.8 ± 0.26 | 27.4 ± 0.22 | 27.7 ± 0.23 | 0.91 |
| BMI category, % |  |  |  |  |  |
| Underweight/normal weight | 30.8 | 31.4 | 31.2 | 29.3 | 0.39 |
| Overweight | 42.9 | 39.7 | 44.0 | 46.4 |  |
| Obese | 26.4 | 28.9 | 24.8 | 24.4 |  |
| Waist circumference, cm^2^ | 97.7 ± 0.38 | 98.1 ± 0.66 | 96.8 ± 0.58 | 98.2 ± 0.61 | 0.77 |
| Systolic blood pressure, mmHg | 125.5 ± 0.59 | 125.8 ± 1.04 | 125.0 ± 0.82 | 125.8 ± 0.89 | 0.92 |
| Diastolic blood pressure, mmHg | 77.1 ± 0.31 | 78.0 ± 0.61 | 76.6 ± 0.55 | 76.4 ± 0.54 | **0.036** |
| Women |  |  |  |  |  |
| *n* | 2562 | 834 | 979 | 749 |  |
| RFS | 7.0 ± 0.10 | 3.6 ± 0.08 | 7.0 ± 0.04 | 10.6 ± 0.10 | **<0.001** |
| Age, y | 46.9 ± 0.30 | 42.4 ± 0.89 | 47.1 ± 0.73 | 51.5 ± 0.82 | **<0.001** |
| Highest level of qualification |  |  |  |  |  |
| Degree | 27.2 | 26.6 | 25.9 | 29.6 | **0.035** |
| Diploma or certificate | 43.0 | 47.9 | 44.1 | 36.2 |  |
| School | 29.8 | 25.5 | 30.1 | 34.3 |  |
| Smoking, % |  |  |  |  |  |
| Current smoker | 14.4 | 22.0 | 13.7 | 6.8 | **<0.001** |
| Ex-smoker | 28.0 | 24.4 | 28.7 | 30.9 |  |
| Never smoked | 57.7 | 53.6 | 57.6 | 62.3 |  |
| Physical activity |  |  |  |  |  |
| Sedentary behavior, min/d | 313 ± 4.0 | 330 ± 11.2 | 309 ± 6.4 | 301 ± 8.5 | 0.06 |
| Meet recommendations, % | 43.9 | 37.1 | 45.6 | 49.1 | **0.005** |
| BMI, kg/m^1^ | 27.0 ± 0.20 | 27.1 ± 0.31 | 27.2 ± 0.33 | 26.6 ± 0.35 | 0.36 |
| BMI category, % |  |  |  |  |  |
| Underweight/normal weight | 44.8 | 45.4 | 41.5 | 48.2 | 0.41 |
| Overweight | 29.6 | 27.3 | 31.8 | 29.2 |  |
| Obese | 25.7 | 27.3 | 26.7 | 22.6 |  |
| Waist circumference, cm^2^ | 87.3 ± 0.47 | 87.0 ± 0.78 | 88.0 ± 0.77 | 86.7 ± 0.79 | 0.95 |
| Systolic blood pressure, mmHg | 119.5 ± 0.46 | 117.4 ± 0.93 | 119.5 ± 0.90 | 121.9 ± 1.11 | **0.001** |
| Diastolic blood pressure, mmHg | 76.0 ± 0.32 | 76.0 ± 0.60 | 75.9 ± 0.55 | 76.1 ± 0.59 | 0.87 |

^1^ Linear regression and chi squared tests were used to test for significant differences between groups in continuous and categorical variables, respectively.

^2^ Data were log-transformed prior to analyses; values represent the geometric mean ± SD

Values represent means ± SD or percentages

**Table S4** Multi-variable-adjusted regression coefficients and 95% CI per 100 unit increase in Dietary Guideline Index and Recommended Food Score for obesity and hypertension-related outcomes in a nationally representative sample of Australian men and women^1^

|  | Men (n=2346) | | Women (n=2562) | |
| --- | --- | --- | --- | --- |
|  | B (95% CI) | P-trend^2^ | B (95% CI) | P-trend^2^ |
| **Dietary Guideline Index** |  |  |  |  |
| BMI^3^ |  |  |  |  |
| Crude | -0.035 (-0.128, 0.058) | 0.45 | 0.024 (-0.072, 0.120) | 0.61 |
| Model 1 | -0.060 (-0.153, 0.034) | 0.23 | -0.024 (-0.120, 0.072) | 0.62 |
| Model 2 | -0.116 (-0.194, -0.037) | **0.004** | -0.046 (-0.136, 0.044) | 0.31 |
| Body weight^3^ |  |  |  |  |
| Crude | -0.017 (-0.117, 0.084) | 0.74 | -0.009 (-0.111, 0.094) | 0.87 |
| Model 1 | -0.022 (-0.124, 0.080) | 0.67 | -0.011 (-0.113, 0.090) | 0.82 |
| Model 2 | -0.088 (-0.173, 0.004) | **0.040** | -0.032 (-0.126, 0.063) | 0.50 |
| Waist circumference^3^ |  |  |  |  |
| Crude | -0.035 (-0.098, 0.028) | 0.27 | 0.021 (-0.050, 0.092) | 0.56 |
| Model 1 | -0.058 (-0.120, 0.005) | 0.07 | -0.042 (-0.111, 0.027) | 0.23 |
| Model 2 | -0.096 (-0.144, 0.049) | **<0.001** | -0.048 (-0.112, 0.017) | 0.15 |
| Systolic blood pressure |  |  |  |  |
| Crude | -10.57 (-19.93, -1.209) | **0.028** | 14.41 (5.856, 22.97) | **0.001** |
| Model 1 | -14.30 (-23.63, -4.974) | **0.003** | -0.476 (-8.365, 7.412) | 0.90 |
| Model 2 | -16.22 (-26.27, -6.176) | **0.002** | 0.609 (-7.394, 8.611) | 0.88 |
| Model 3 | -14.59 (-24.81, -4.377) | **0.006** | 1.392 (-6.186, 8.969) | 0.72 |
| Diastolic blood pressure |  |  |  |  |
| Crude | -4.279 (-8.571, 0.013) | 0.051 | 2.666 (-2.795, 8.127) | 0.33 |
| Model 1 | -6.190 (-10.52, -1.863) | **0.006** | -0.844 (-5.544, 5.375) | 0.98 |
| Model 2 | --5.922 (-10.35, -1.492) | **0.010** | 0.627 (-5.084, 6.338) | 0.83 |
| Model 3 | -3.647 (-8.335, 1.041) | 0.13 | 1.448 (-4.004, 6.901) | 0.60 |
| **Recommended Food score** | | | | |
| BMI^3^ |  |  |  |  |
| Crude | 0.031 (-0.274, 0.337) | 0.84 | -0.340 (-0.720, 0.040) | 0.08 |
| Model 1 | 0.286 (-0.618, 0.047) | 0.091 | -0.622 (-0.967, -0.278) | **0.001** |
| Model 2 | -0.119 (-0.435, 0.198) | 0.456 | -0.144 (-0.514, -0.226) | 0.44 |
| Body weight^3^ |  |  |  |  |
| Crude | 0.071 (-0.281, 0.423) | 0.69 | -0.412 (-0.822, -0.001) | **0.049** |
| Model 1 | -0.021 (-0.402, 0.359) | 0.91 | -0.457 (-0.859, -0.054) | **0.027** |
| Model 2 | 0.145 (-0.253, 0.543) | 0.47 | -0.101 (-0.291, 0.493) | 0.61 |
| Waist circumference^3^ |  |  |  |  |
| Crude | 0.027 (-0.208, 0.262) | 0.82 | -0.155 (-0.469, 0.160) | 0.33 |
| Model 1 | -0.328 (-0.570, -0.086) | **0.009** | -0.487 (-0.759, -0.215) | **0.001** |
| Model 2 | -0.218 (-0.469, -0.034) | 0.09 | -0.153 (-0.440, -0.134) | 0.29 |
| Systolic blood pressure |  |  |  |  |
| Crude | -14.55 (-56.49, 27.38) | **0.490** | 73.29 (41.57, 105.0) | **<0.001** |
| Model 1 | -68.12 (-110.6, -25.62) | **0.002** | -3.225 (-31.83, 25.33) | 0.82 |
| Model 2 | -69.05 (-118.3, -198.4) | **0.007** | 2.734 (-28.64, 34.11) | 0.86 |
| Model 3 | -67.25 (-116.7, -17.82) | **0.009** | 5.176 (-25.50, 35.85) | 0.74 |
| Diastolic blood pressure |  |  |  |  |
| Crude | -25.31 (-47.04, -35.81) | **0.023** | 5.265 (-14.75, 25.28) | 0.60 |
| Model 1 | -46.89 (-68.29, -25.49) | **<0.001** | -8.593 (-29.48, 12.29) | 0.414 |
| Model 2 | -36.80 (-59.7,-13.9) | **0.002** | -1.733 (-25.58, 21.11) | 0.88 |
| Model 3 | -34.46 (-57.91, -10.98) | **0.005** | 0.826 (-20.25, 21.90) | 0.94 |

^1^ All coefficients are presented as x100 multiple of the original coefficient.

^2^ Data were analyzed using multiple linear regression. Model 1: adjusted for age and education, Model 2: additionally adjusted for smoking, physical activity, urban or rural location, female life stage (women only), energy intake misreporting and information on whether a participant was on a diet and whether their dietary recall was typical of their habitual consumption; Model 3: additionally adjusted for BMI.

^3^ Data were log-transformed prior to analysis
